# Design of a Hybrid Inertial and Magnetophoretic Microfluidic Device for CTCs Separation from Blood

**DOI:** 10.3390/mi12080877

**Published:** 2021-07-26

**Authors:** Rohollah Nasiri, Amir Shamloo, Javad Akbari

**Affiliations:** Department of Mechanical Engineering, Sharif University of Technology, Tehran 11365-11155, Iran; Rhnasiri90@gmail.com (R.N.); Akbari@sharif.edu (J.A.)

**Keywords:** microfluidics, cell separation, CTCs, inertial, magnetophoretic, nanoparticle

## Abstract

Circulating tumor cells (CTCs) isolation from a blood sample plays an important role in cancer diagnosis and treatment. Microfluidics offers a great potential for cancer cell separation from the blood. Among the microfluidic-based methods for CTC separation, the inertial method as a passive method and magnetic method as an active method are two efficient well-established methods. Here, we investigated the combination of these two methods to separate CTCs from a blood sample in a single chip. Firstly, numerical simulations were performed to analyze the fluid flow within the proposed channel, and the particle trajectories within the inertial cell separation unit were investigated to determine/predict the particle trajectories within the inertial channel in the presence of fluid dynamic forces. Then, the designed device was fabricated using the soft-lithography technique. Later, the CTCs were conjugated with magnetic nanoparticles and Ep-CAM antibodies to improve the magnetic susceptibility of the cells in the presence of a magnetic field by using neodymium permanent magnets of 0.51 T. A diluted blood sample containing nanoparticle-conjugated CTCs was injected into the device at different flow rates to analyze its performance. It was found that the flow rate of 1000 µL/min resulted in the highest recovery rate and purity of ~95% and ~93% for CTCs, respectively.

## 1. Introduction

Circulating tumor cells (CTCs) captured from blood samples have great potential for the diagnosis and treatment of cancer. Microfluidics has emerged as a great technology for cell separation application by exploiting different properties of cells and different physical principles [1,2]. These techniques are categorized as microfluidic microfilters, deterministic lateral displacement, hydrodynamic filtration, inertial methods as well as methods based on external force fields such as dielectrophoretic (DEP), acoustic, magnetic, and optical separation methods [3,4]. Additionally, these methods can be categorized into two groups of label-free and affinity-based/labelled approaches. Label-free cell separation methods function based on the inherent cell properties (e.g., density, size, shape, deformability, electrical properties, magnetic susceptibility, compressibility, refraction index) while affinity-based cell separation approaches use “labels” (e.g., particle-antibody conjugates to a membrane protein specifically) to capture target cells [5]. Among the abovementioned microfluidic methods for cell separation, inertial methods from passive methods and magnetic methods from active methods offer great potential for different target cell separation. Moreover, these methods offer merits of efficiency, biocompatibility, and simple configurations [6].

Inertial microfluidic devices benefit from fluid inertia i.e., fluid dynamic forces for cell sorting applications. In this method, particle movement is affected by inertia and they do not follow streamlines of flow as in other hydrodynamic-based cell separation methods [7,8,9]. Cells/particles in a straight microchannel within a fluid flow experience a lift force because of the balance between shear-gradient induced and wall-induced lift forces, called inertial lift force (Equation (1)) [10] and this force is dependent on the size of the particle, Reynolds number (Equation (2)), and position of particles within the cross-section of the channel [9,11].

(1) $$F_{L}=\frac{\rho_{f}U_{m}^{2}d_{p}^{4}{}_{\;}}{D_{h}^{2}}f_{L}\left(Re_{C},z\right),$$

(2) $$Re_{C}=\frac{\rho_{f}U_{m}D_{h}}{\mu_{f}},$$

(3) $$D_{h}=\frac{2W\times H}{W+H},$$

In the above equations, $\rho_{f}$, $\mu_{f}$, and $U_{m}$ are density of fluid, fluid viscosity, and average velocity of fluid flow, respectively. Moreover, *D_h_* represents the hydraulic diameter of the microchannel that can be calculated using Equation (3), in which W and H represent the width and height of the channel, respectively. d_p_ is the size of the particle, and f_L_ represents the coefficient for inertial lift force, which is dependent on the particle position within the channel cross-section (*z*) and Reynolds number. Curvature in the channel design and presence of the obstacles in the path of fluid flow within the microchannel leads to the formation of secondary flows, called Dean flows. The creation of the secondary flow exerts a drag force on the particles (*F_D_*), as it is expressed in Equation (4) and is called the Dean drag force. The competition between F_L_ and F_D_ in inertial microfluidic channels for cell separation determines the equilibrium locations of the cells/particles [12]. In Equation (4), *U_Dean_* represents the secondary flow intensity (velocity).

(4) $$F_{D}=3\pi \mu U_{Dean}d_{p},$$

Inertial microfluidic devices were widely used in previous studies for cell separation using different geometries for the channel. Among them, the spiral [13], contraction-expansion [14], and serpentine channels [15] are the most common geometries for target cell separation. More examples of inertial-based cell separation were reviewed in our recent review paper [12].

The magnetic cell separation method as an active method was introduced by Molday et al. [16] and is one of the typical non-invasive approaches for cell separation in biomedical research. Magnetophoresis represents the controlled manipulation and separation of cells from a mixture of cells by using a magnetic force field.

The magnetic force ($F_{m}$) that is exerted to a magnetic particle with the volume of *V_p_* in the presence of a magnetic field with a strength of $\vec{B}$ can be determined using the following equation [17]:

(5) $${\vec{F}}_{m}=\frac{V_{p}\left(\chi_{p}-\chi_{f}\right)}{\mu_{0}}(\vec{B}\cdot \nabla )\vec{B}$$

where $\chi_{p}$ and $\chi_{f}$ represents magnetic susceptibilities of the particle and the carrier fluid (medium), respectively, and $\mu_{0}$ represents the permeability of vacuum ($4\pi \times 10^{-7}{\mathrm{TmA}}^{-1}$). Based on Equation (5), the key parameters in magnetic manipulation of the cells within microfluidic device are the magnetic gradient ($\nabla \vec{B}$), the particle volume ($V_{p}$), and magnetic susceptibility of particle ($\chi_{p}$), and the carrier flow magnetic susceptibility ($\chi_{f}$) for designing an effective magnetophoretic cell separation device [18]. To improve the susceptibility difference (Δχ), target cells can be bonded with magnetic particles and specific antibodies. Micro/nanomagnetic particles are synthesized and conjugated with antibodies to attach to specific cell surface antigens. Selecting a non-toxic material for magnetic particles is crucial. Iron oxides of magnetite Fe_3_O_4_ and maghemite *γ*-Fe_2_O_3_ are extensively used for this purpose [19,20].

CTCs are target cells of interest in magnetic separation. For instance, Plouffe et al. [21] used electromagnets and magnetic microbeads in a microfluidic chip to capture MCF-7 breast cancer cells from whole blood with an efficiency of ~85–95% and purity of ~90–55%.

In cases of heterogeneous and complex samples, such as a blood sample with rare CTCs, employing only a single module of cell separation is challenging. Hence, the integration of cell separation methods can solve the limitation of the single microfluidic module for cell separation to attain higher separation efficiency or purity and improving the throughput by exploiting multiple cellular properties [22,23]. A hybrid cell separation microfluidic platform that integrates two different modules of cell separation including passive and active methods could provide a superior performance in target cell isolation, as it takes the advantages of the higher accuracy of active cell separation approaches and the higher throughput of passive techniques [24]. For example, Zhang et al., [25] proposed a hybrid device by integration of a DEP method with an inertial microfluidic device that employed dielectrophoretic force as an external force field in active separation of cells and integrated it with inertial method for particle manipulation. In another study, Zhou et al. [26] reported the design of a hybrid microfluidic platform by integrating a high throughput inertial cell separator and an acoustic cell sorter to achieve to higher accuracy of single-cell separation. Magnetophoresis-based cell separation can be a superior option for applications in a low-cost and low-resource setup, as the magnetic field could be created by simple permanent magnets for cell manipulation. Toner et al. [27] proposed an integrated microfluidic device called CTC-iChip, which included a DLD separation unit and inertial focusing channel as well as a magnetophoresis-based cell separation module for capturing CTCs from blood cells. However, the first stage of their proposed device i.e., the DLD separator had clogging problems and restricted the throughput of their proposed hybrid device. Recently, Huang et al., proposed a combined microfluidic device called i-Mag device, composed of three units including an inertial separator for RBC separation, inertial focusing and magnetic cell separator for CTCs separation from WBCs. In this device, the WBCs tagged to magnetic nanoparticles and CTCs were separated in an antigen-independent manner [24]. They reached a separation efficiency of ~94% and purity of 93.6% for separation of CTCs from diluted blood.

In this work, the benefits of both inertial and magnetic cell separation approaches are used to separate cancer cells from blood with higher purity compared to the single inertial device. Since some of the CTCs and WBCs have similar sizes and the inertial method is a size-based cell sepration approach, it has limitations in capturing tumor cells of similar size to WBCs with high purity. So, the combination of both passive (inertial) and active (magnetic) approaches for cell separation can address this issue and can provide a better performance for the separation of rare cells by benefiting from the high precision of active methods and the high throughput of passive techniques. Therefore, in this work, MCF-7 cells are conjugated with Ep-CAM antibodies and magnetic nanoparticles to improve their magnetic susceptibility, and the mixture of these cells with blood cells is injected into our proposed hybrid device for cell separation. In the first stage, an asymmetric serpentine inertial microfluidic device is designed to remove the majority of RBCs and WBCs, by employing inertial lift forces and dean drag force to sort cells based on their sizes. In the asymmetric serpentine inertial cell separator the smaller size of cells i.e., RBCs and WBCs can be focused in the sidewalls of the channel while the larger cells i.e., CTCs and some of the WBCs which have a similar size to CTCs can be focused in the middle of the channel. So, using magnetically labeled CTCs and a permanent magnet in second cell separator stage of the device, the CTCs can be captured by magnetic cell separator with a higher purity compared to the single inertial channel by eliminating WBCs. By using permanent magnets next to a magnetophoretic cell separation section followed by a focusing region, the CTCs that were conjugated with magnetic nanoparticles are separated from remaining blood cells in the presence of the magnetic field. Although the single module of inertial cell separation and magnetic cell separation was investigated extensively by different research groups, only there are a few studies about the combination of the mentioned methods which are reviewed in recent review papers [12,22]. Compared to other works for the combination of inertial and magnetic methods, our platform has a linear structure that can be fabricated in arrays of these chips in a parallel patterns to achieve high-throughput target cell separation by having one inlet for all parallel channels that enables high-throughput CTCs separation for clinical applications. Moreover, because a high-throughput serpentine inertial microfluidic was used in the first-stage, our proposed hybrid device does not have clogging issues and has a simple structure compared to the hybrid devices which use the DLD method for blood cell capturing.

## 2. Materials and Methods

### 2.1. Description of the Proposed Hybrid Device

**Figure 1:**
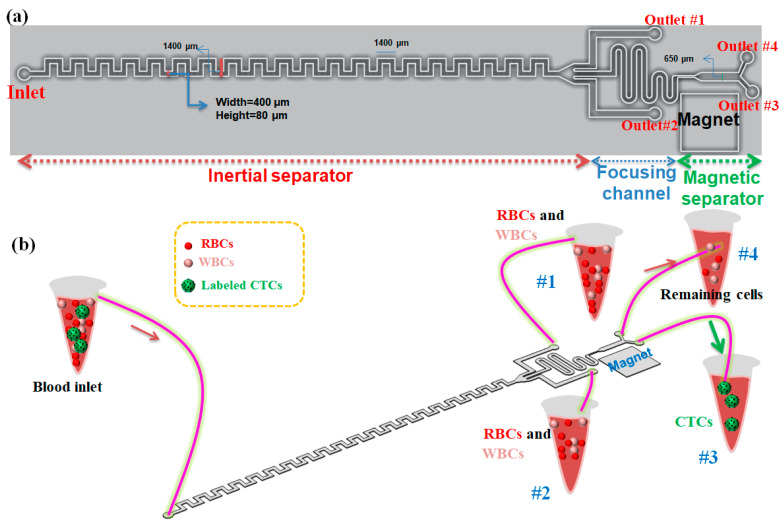
(**a**) 2D schematic of our proposed hybrid microfluidic cell separation devcie in optimal condition; (**b**) 3D schematic of the proposed hybrid device composed of three units for labeled CTCs separation from blood. The device has 4 outlets (#1 and #2 are outlets for inertial section, #3 and #4 are outlets for magnetic section).

The schematic of our proposed hybrid device including an asymmetric serpentine inertial channel, an inertial focusing channel, and a magnetic cell separation device for CTC separation from whole blood is shown in Figure 1a. As it is shown, this device has one inlet for sample injection into the device and four outlets for collecting the outlet samples. Two side outlets in the inertial step are designed for collecting non-target cells including RBSs and WBCs (in optimal condition for inertial section), and two outlets in the magnetic section of the device are for capturing CTCs due to the presence of a magnetic field in the outlet close to the magnet and collecting non-target cells and nanoparticles in another outlet. Cells flowing in the inertial section of our device will not only experience the inertial lift force but also simultaneously feel the Dean drag force originated by the secondary flow. To perform cell focusing/sorting at specific flow rates, one criterion concerning the particle confinement ratio (CR = d_p_/D_h_) should satisfy CR > 0.07 condition [28]. Based on this criterion, the width and the height of the inertial channel were chosen to be 400 and 80 μm, respectively, to ensure the separation of large-sized CTCs from smaller cells (RBCs and WBCs).

In this paper, we present a three-stage cell separation chip that integrates the high-throughput inertial asymmetric serpentine microfluidic module with a precise active magnetophoresis-based cell separation approach to achieve fast, accurate, and affinity-based (labeled) CTC separation. The first-stage serpentine inertial sorter is employed to quickly eliminate the RBCs and majority of the WBCs and collect them in outlets #1 and #2, while the other serpentine inertial focuser and the magnetic separator are placed and designed as the second and third segments, respectively, to eliminate the remaining WBCs and capture magnetically labeled CTCs in a precise manner. So, CTCs which are conjugated with magnetic nanoparticles are attracted to the target collecting outlet (#3) via the magnetic force, while the other non-target cells move along their paths towards the non-target outlet (#4). Additionally, both separation units, as well as focusing sections, are integrated in a single microchip, which prevents cell loss during cell transfer in case by using tubing connection between the distinct modules (Figure 1b). Although the asymmetric serpentine inertial cell separation module can remove the RBCs and WBCs to capture with good performance, to improve the separation efficiency and purity of separated CTCs, the inertial unit is connected to a magnetophoretic cell separation unit to separate CTCs in a specific antigen-dependent manner. For this purpose, the CTCs are conjugated with antibodies and nanoparticles to improve their magnetic susceptibility and the conjugation process will be discussed in the following sections. Aligning the cells to the same string is important to attain efficient magnetic cell separation. For this purpose, a serpentine inertial focuser was introduced before the magnetic cell sorter section to focus cells into the same string in presence of the inertial effects. Additionally, the width of the channel in the magnetic cell separation unit is expanded to reduce the velocity of cells to achieve effective magnetophoretic force on cells. To generate a non-uniform magnetic field for deflecting the magnetic nanoparticle-labeled CTCs, three NdFeB N50 permanent magnets with a size of 6 mm × 4 mm × 1.5 mm were placed next to the magnetic channel (~1 mm distance from the channel).

### 2.2. Numerical Simulation

To have an insight into the fluid flow behavior within the channel, numerical simulations were performed to analyze the fluid flow within the proposed hybrid device. To simulate the fluid flow within the proposed channel, governing equations including Navier–Stokes and continuity equations for momentum and mass conservation (Equations (6) and (7)) were solved with our lab-made finite element method (FEM) solver. This solver employs the Affine Invariant Adaptive Newton Codes. Pseudo-time-stepping stabilizes the solution and results in convergence of the solution by adjusting the Courant–Friedrichs–Lewy (CFL) number. The constant flow rate was considered as a boundary condition for the inlet of the device and atmospheric pressure was considered as a boundary condition for outlets of the channel. To convert the nonlinear governing equation to a system of ordinary equations the Galerkin scheme was used. For numerical simulation, unstructured tetrahedral grids with sizes from 1.6 μm up to 6.1 μm were generated.

(6) $$\rho_{{}_{f}}\left(\vec{u}\cdot \nabla \right)\vec{u}=\mu_{f}\nabla^{2}\vec{u}-\nabla P,$$

(7) $$\nabla \cdot \vec{u}=0,$$

The dilution of blood samples is crucial for the efficient performance of the inertial cell separation to avoid cell–cell interactions to enable the inertial lift forces to be effective in cell sorting and in most of the previous studies the blood samples were diluted before processing within the channel [29]. Since in this study the blood sample is diluted 20 times with phosphate-buffered saline (PBS), by considering the hematocrit of the whole blood sample to be 45%, therefore by 20 times dilution of the whole blood, about 95% volume of the diluted blood would be the PBS buffer and by considering the density of whole blood and PBS as 1060 kg/m^3^ and 1000 kg/m^3^ respectively, the density of diluted blood sample will be ~1003 kg/m^3^.

In order to predict the role of the inertial cell separator in our hybrid device, the particles/cells’ trajectories in the inertial section of our proposed device were investigated numerically at different flow rates. For particle tracing, the transient equation of motion was solved with a 0.00002 s time step (Equation (8)).

(8) $$\frac{d(m_{p}v_{p})}{dt}=F_{total}=F_{\mathrm{Inertial}\;\mathrm{Lift}}+F_{Drag}+F_{\mathrm{Added}\;\mathrm{mass}}+F_{Saffman},$$

In the above equation, $m_{p}$ and $v_{p}$ are the particles’ mass and the velocity, respectively. According to this equation, the inertial lift force, added mass lift force, and Saffman lift force and drag force (viscous drag force of main flow and Dean drag) are applied to particles as driving and effective forces. For viscous drag force, we employed the proposed equation by Khan et al. [30] as the following equation.

(9) Fdrag=π4dp2ρfvt2(1.84Re′−0.31+0.293Re′0.06)3.45,

(10) Re′=ρfvtdpμ,

(11) $$v_{t}=u-v_{p},$$

In which $v_{t}$ represents the relative velocity of the fluid to the particle. Saffman lift and added mass forces are applied to the particles according to the following equations [31]:

(12) $$F_{Saffman}=20.3d_{p}^{2}{\left(\gamma \upsilon^{-1}\right)}^{0.5}\left(\vec{u}-{\vec{v}}_{p}\right)$$

(13) $$F_{\mathrm{Added}\;\mathrm{mass}}=\frac{\pi }{12}\rho_{f}d_{p}^{3}\frac{d}{dt}\left(\vec{u}-{\vec{v}}_{p}\right),$$

In the above equations, γ is the shear rate and υ is the kinetic viscosity. We used the proposed modified formulation by Liu et al., [32] for applying inertial lift force. More details about this formulation were reported in our previous work [1]. Three types of particles with sizes of 6 µm, 10 µm, and 15 µm were used as average sizes of RBCs, WBCs, and CTCs, respectively. All types of particles are distributed randomly at the inlet of the inertial channel and after injection into the channel they experience fluid dynamic forces including lift force and drag force which are dependent on the particles’ sizes, so by moving forward within the channel by the viscous drag force, and due to the dominant lift force (inertial lift force) and dean drag force due to the secondary flow within the cross-section of the channel, each size of the particles can be focused into distinct equilibrium positions at the end of the inertial cell sorter which enables the separation of particles with different sizes.

### 2.3. Viscosity Measurement for Diluted Blood

We performed a rheology test at different shear rates to determine the viscosity of the diluted blood sample. A diluted blood sample containing 200 µL whole blood and 4 mL PBS was mixed and the viscosity of the mixture was measured using a rotational viscometer (Brookfield, Waukesha, WI, USA) at different shear rates and at constant temperature (20 °C). Figure S1 shows the viscosity change versus shear rate for the 20 times diluted blood sample; it is concluded that by changing the shear rate the value of the viscosity does not change significantly, so the 20 times diluted blood with PBS behaves as a Newtonian fluid with a viscosity of ~1.12 mPa.s at 20 °C. Therefore, this value is considered in our numerical simulation for fluid flow within the channel.

### 2.4. Fabrication of the Device

After designing the proposed channel using AutoCAD (version 2017), the photomask was printed on high-resolution film, as shown in Figure S2a then standard soft photolithography was used to fabricate the proposed hybrid device. Briefly, a 3-inch silicon wafer was used as a substrate for mold, Su-8 2050 photoresist (Microchem) was used to spin coat on the wafer based on the Microchem data sheet (500 rpm, 10 s, 100 rpm/s^2^, 1800 rpm, 30 s, 300 rpm/s^2^) to achieve to 80 µm thickness. This was then baked on a hot-plate at 65 °C for 3 min and another 8 min at 95 °C as soft bake. The silicon wafer was exposed to UV light (1.5 mW/cm^2^) for 60 s using a mask aligner, and post-exposure baked at 65 °C for 3 min and another 8 min at 95 °C. Then, the spin-coated wafer was immersed in developer solution (Microchem) to remove the unwanted section of the mold. To develop the microstructure, the sample was immersed in a photoresist developer for 5 min and washed three times by isopropyl alcohol. All chips were then dried with filtered nitrogen before being baked for 2 min at 150 °C to ensure the stability of SU-8. After preparing the mold as Figure S2b, a mixture of polydimethylsiloxane (PDMS) and curing agent with a weight ratio of 10:1 was prepared, and by proper mixing and desiccating using a desiccator for 60 min the bubbles were removed, then the mixture poured on the mold and after curing in 80 °C oven for 2 h, the PDMS was separated from the mold. Later, the inlet and outlets of the channel were created via the flat-tip puncher. Then, the open PDMS channel and a glass slide placed in plasma cleaner (400 mTorr) for 2 min to bond them together permanently to complete the fabrication process as shown in Figure S2c.

### 2.5. Nanoparticles Synthesis and Conjugation of Antibody-Nanoparticle with Cells

To Capture the CTCs in the magnetic cell separation section in our proposed device, the CTCs (MCF-7 cells) should be tagged to magnetic nanoparticles to improve their magnetic susceptibility in presence of the magnetic field as it was described in our previous work [33]. For this purpose, Magnetite nanoparticles (Fe_3_O_4_) were synthesized using the coprecipitation technique [33,34] and then conjugated with CTCs via antigen–antibody complexes. To prepare the nanoparticles, reagents of FeCl_3_·6H_2_O (6.875 g) and FeCl_2_·4H_2_O (2.237 g) as raw materials were dissolved in 20 mL of deionized (DI) water in a constant magnetic stirring condition for 45 min and in a nitrogen atmosphere. Later, 1.5 g of arginine (C_6_H_14_N_4_O_2_) reagent as the pH control agent was dissolved in 10 mL DI water and was added to the abovementioned solution under magnetic stirring to achieve pH 9. Then, the reaction proceeded for 45 min with constant stirring under a nitrogen atmosphere. Lastly, the precipitates were washed repeatedly with DI water and ethanol and then dried at 70 °C in a vacuum desiccator for 5 h to obtain the Fe_3_O_4_ magnetic nanoparticles.

### 2.6. Cell Culture

MCF-7 cells were cultivated in a 75 cm^2^ flask in an incubator with 37 °C, 5% CO2, and 80% relative humidity conditions. Dulbecco’s Modified Eagle Medium (DMEM) containing 10% fetal bovine serum (FBS) and 1% Penicillin-Streptomycin (pen-strep) was employed as the cell culture medium and the media was changed every other day. After reaching 80% confluency, the cells were trypsinized and detached from the flask, and suspended in phosphate-buffered saline (PBS). Whole blood from a healthy donor in a vial which was coated with anticoagulant reagent (EDTA) was used and diluted 20 times with PBS to avoid cell–cell interactions after injection into the channel, in which the dilution of blood sample is crucial for efficient sorting of cells. The MCF-7 cells were conjugated with nanoparticles (based on the protocol that we will discuss in the following section) and mixed with blood cells in a 1:1000 cell ratio. To quantify the cell numbers at outlets, the target cells (MCF7) were stained using DAPI staining to help for visualization of them after separation. For this purpose, first, the cells were fixed with paraformaldehyde (PFA) (4%). Additionally, 0.1% Triton X-100 in PBS was utilized to permeabilize the cells, and later the cells were stained with DAPI.

### 2.7. Experimental Setup

To test the performance of our fabricated device, a fluorescence microscope, a syringe pump for sample injection, collecting tubes, tubing, 10 mL syringes, and a camera were used, as shown in Figure S3a. By tuning the flow rate of the sample injection into the device using a syringe pump, the outlet flows were collected in glass vials, as shown in Figure S3b, for further analysis to evaluate the device performance in terms of recovery rate (separation efficiency) for target cells as well as purity of the separated cells.

## 3. Results and Discussion

**Figure 2:**
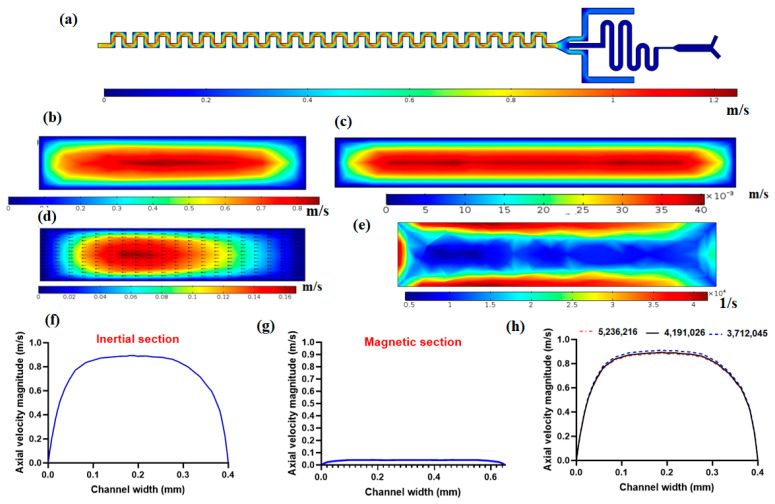
Fluid flow simulation within the proposed hybrid channel at 1000 µL/min. (**a**) The velocity magnitude at mid-plane for the hybrid device; (**b**) axial velocity magnitude within the cross-section of the inertial cell separator; (**c**) axial velocity magnitude in the cross-section of magnetic cell separator section; (**d**) Secondary flow distribution and vectors in cross-section of inertial separator; (**e**) Shear rate in the cross-section of the inertial separator; (**f**) axial velocity profile in inertial cell separator; (**g**) axial velocity profile in magnetic cell separator; (**h**) mesh independency analysis for axial velocity magnitude.

Fluid flow simulation was performed to solve the governing equations by considering the inlet and outlet boundary conditions and wall boundaries. Figure 2a shows the velocity field in the mid-plane of the channel. As it is demonstrated in this figure, near the walls due to no-slip conditions on walls, there is zero velocity and at the middle of the channel, there is the maximum velocity because of the parabolic velocity distribution within the channel in laminar flow. Figure 2b shows the axial velocity magnitude in the cross-section of the channel. As it is shown in this figure, the maximum velocity occurs in the central region of the cross-section, and the minimum velocity occurs near the walls due to the no-slip condition on the walls. As mentioned in the above section, the magnetic separator has a larger channel width (650 µm) to reduce the velocity of flow so as to have effective magnetic force on nanoparticle conjugated CTCs in the presence of the magnetic field. Figure 2c shows the velocity distribution in the magnetic cell separator cross-section, which shows the lower values of velocity in the magnetic section compared to the inertial separator. Another important aspect of the proposed channel for inertial cell separation is the formation of secondary flow within the cross-section of the channel, as shown in Figure 2d. This secondary flow plays a vital role in helping lateral migration of particles/cells, which in its competition with inertial lift force leads to distinct equilibrium positions for particles based on their size. Figure 2e shows the shear rate in the cross-section of the inertial channel, as shown in this figure, the shear rate is at its maximum near the walls and minimum far away from walls. The shear rate plays a crucial role in inertial lift force for the separation of cells. In Figure 2f,g, the axial velocity profiles for inertial separator and magnetic cell separator are plotted, respectively. These velocity profiles are along a mid-line in the direction of the width of the channel in a cross-section of the channel. As shown in Figure 2f,g, the magnetic cell separation section has significantly lower values of velocity compared to the inertial section, which is important for effective magnetic force on target cells that are labeled with magnetic nanoparticles. Moreover, we performed mesh independency analysis with different numbers of elements for velocity profiles for axial velocity, as shown in Figure 2h, and after choosing 4,191,026 meshes with a minimum size of 1.6 µm and maximum size of 6.1 µm, we can see that the velocity profile is independent of the number of elements for the computational domain.

**Figure 3:**
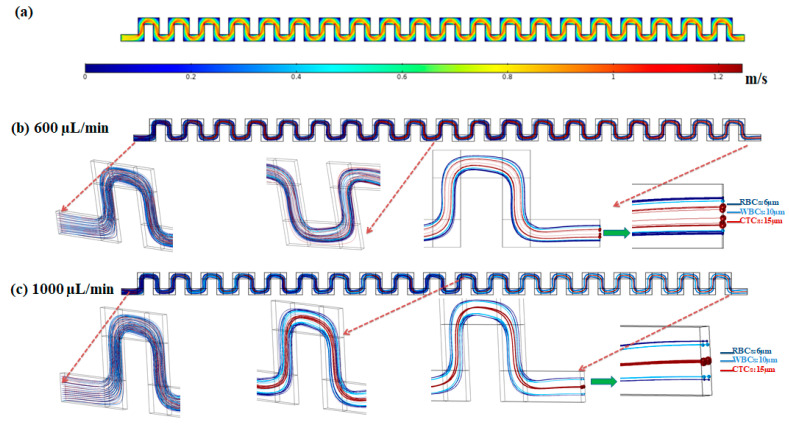
(**a**) Velocity distribution in the mid-plane of the inertial section of the channel at 1000 µL/min. (**b**,**c**) Particle trajectories at flow rates of 600 and 1000 µL/min in the inertial section of the proposed hybrid device.

In order to show the capability of the inertial section of the proposed hybrid device for blood cells (RBCs and WBCs) separation, simulation of fluid flow and particle tracing were performed for the inertial section. After solving the governing equations for fluid flow in this channel, numerical simulation for particles’ movement within the channel was performed and by applying effective forces on particles at specific flow rates, particle trajectories were determined. Figure 3 shows the velocity field within the mid-plane of the inertial section of the channel at flow rate of 1000 µL/min.

The effective forces on particles, including the inertial lift force, drag force, added mass force, and saffman lift force are applied to the equation of motion for particles in the Lagrangian approach. To simulate the particle trajectories within the inertial section of our proposed device, separation of three different particles with sizes of 6, 10 and 15 µm corresponding to the approximate average size for RBCs, WBCs, and CTCs, respectively, were investigated. The density of all cells/particles was considered to be 1060 kg/m^3^. Due to the dilution of the blood sample in the current study and most inertial cell separation approaches, it was assumed that the particle–particle interactions are negligible. The simulations were conducted at flow rates of 600 and 1000 µL/min. The particles contained by the fluid (blood sample) entered from the inlet and passed through the serpentine inertial channel. Based on effective applied forces, the particles were focused and sorted based on their size, into distinct positions at the outlet of the serpentine inertial channel. As shown in Figure 3b, at a low flow rate (600 µL/min) the equilibrium positions of the three different particles are close to each other. By increasing the flow rate to 1000 µL/min, the particles are focused in three different equilibrium positions which enable the separation of particles from each other (Figure 3b). As shown in the particle tracking results for particle trajectories within the inertial serpentine channel, blood cells are focused in the sidewalls of the channel and can be captured in the inertial channel. By going forward within the channel, the randomly distributed particles at the inlet of the channel experience different values of the inertial lift force, and drag force and at the end of the channel are focused into distinct equilibrium positions, with their equilibrium position depending on their size and also the flow rate. Our simulation for the particle trajectories in the inertial section of our proposed device demonstrates that in the first module (inertial section) of our proposed hybrid device, the blood cells i.e., RBCs and WBCs can be depleted (captured by inertial section) and larger cells i.e., CTCs or some WBCs which overlap in size with CTCs, can be entered into the second separation module (magnetic cell separation section) which enables a high separation efficiency and purity of CTCs from the remaining cells.

**Figure 4:**
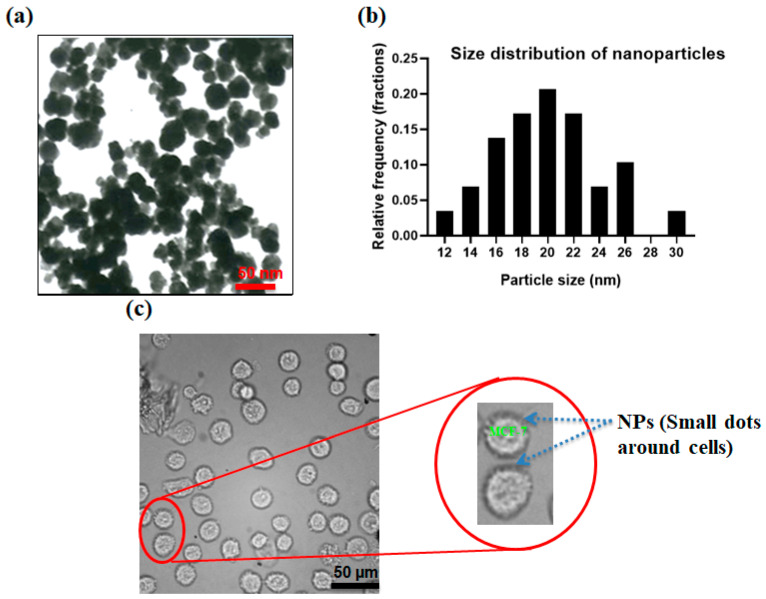
(**a**) Nanoparticles; (**b**) size distribution for NPs; (**c**) optical image of MCF-7 which are surrounded by antibody-conjugated nanoparticles.

Figure 4a shows the transmission electron microscopy (TEM) image of produced nanoparticles. Figure 4b shows the size distribution of the produced nanoparticles which the size of the nanoparticles was analyzed using ImageJ software and sizes in the range of 10–30 nm. By the addition of specific interfering reagents, these nanoparticles can be conjugated with the Ep-CAM antibodies and later they can specifically attach to the antigens on MCF-7 cells. Arginine, as an α-amino acid containing two carboxyl and amine groups, can confine magnetite nanoparticles via a shell-core binding and the carboxyl groups are placed outwards and can be attached to the amine group of the antibodies. N-Hydroxysuccinimide (NHS) and 1-Ethyl-3-dimethylaminopropyl (EDC) were employed to activate the functional groups and make the conjugation of the nanoparticle–antibody. After preparing the antibody-conjugated nanoparticles in PBS buffer, 100 µL of this solution were mixed with 1 M MCF-7 cells in PBS and incubated for 1 h to enable the conjugation of the MCF-7 cells to nanoparticles. Figure 4c shows the binding of antibody-conjugated nanoparticles to the MCF-7 cells. As shown in Figure 4c, CTC membranes are surrounded by magnetite nanoparticles to increase the magnetic susceptibility of the cells in the presence of the magnetic field.

**Figure 5:**
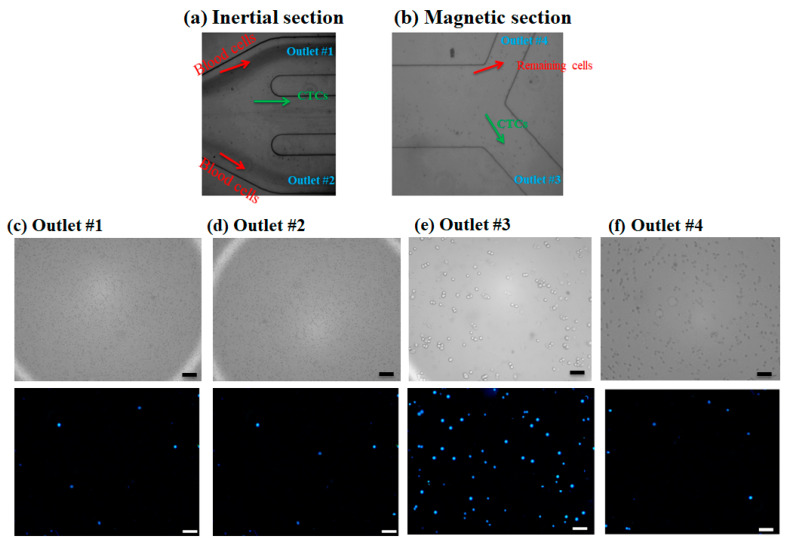
(**a**,**b**) Microscopic images for cell suspension flow at the outlet of inertial and magnetic separation, respectively. (**c**–**f**) Brightfield image and DAPI stained image for MCF-7 cells in different collecting outlets in inertial and magnetic separator. Scale bares are 100 µm.

The injection of the prepared diluted blood sample containing CTCs was processed within the channel without any clogging issues, and dilution of the blood sample aids the clogging-free and minimal cell–cell interactions for the efficient performance of the proposed hybrid device. After performing experiments for blood samples containing nanoparticle conjugated CTCs in different flow rates by injecting the sample into the device and collecting the samples in glass vials for each outlet, specific volumes of each sample were examined using a fluorescence microscope and the number of cells was counted with ImageJ software. As mentioned in the above sections, to distinguish CTCs from non-target cells (blood cells), a cell staining technique was employed. For this purpose, DAPI fluorescent dye was used to stain the CTCs, and using fluorescence microscopy, two matching bright field and fluorescence images were captured from the collected samples in each outlet chamber. In a bright-field image, both target cells and non-target cells can be observed, while in a fluorescence image only the CTCs can be observed with a blue color. Thus, the CTCs can be distinguished from other cells. Figure 5a,b shows microscopic images for the outlets of the inertial section and magnetic cell separator section, respectively. As shown in Figure 5a, the majority of blood cells are directed into the outlets 1 and 2, which was confirmed by a small number of the stained CTCs cells in these outlets (Figure 5c,d) and larger cells i.e., CTCs and some of the blood cells are entered into the magnetic cell separator which are collected in outlet 3 and outlet 4. Figure 5c,d shows the bright field and fluorescent images for the collected samples in outlets 1 and 2 for the inertial section of the hybrid device, Figure 5e shows the bright field and fluorescent image for the collected sample in outlet 3 (the target outlet for CTC isolation in the magnetic separator), and Figure 5f shows the non-target outlet (in magnetic separator for collecting the remaining cells) at 1000 µL/min, respectively. As shown in these images, the inertial section is able to deplete ~100% of blood cells; also in this section we have minimal numbers of collected CTCs in outlets 1 and 2, and most of the CTCs are entered into the magnetic cell separator section which are captured by magnetic field and collected in outlet 3. The fluorescent images for CTCs at different outlets and flow rates are shown in Figure S4. Because the majority of CTCs are captured by magnets and are collected in outlet 3, which is next to the magnet, this confirms the effective bonding of nanoparticles to CTCs for different flow rates.

To have a quantification on the separation efficiency and purity of separated cells, ImageJ software was used to count the stained and non-stained cells and have the number of cells in the inlet sample the separation efficiency and purity calculated. The ratio of the number of CTCs that exist in the particular outlet to the total number of CTCs in the injection process at the inlet is defined as the recovery rate/separation efficiency (Equation (14)) and this factor was calculated for each flow rate. Additionally, the purity of isolated target cells is defined as the ratio of the number of CTCs in a particular collecting chamber to the entire number of cells in that chamber as Equation (15).

(14) $$\mathrm{Recovery}\;\mathrm{rate}=\frac{\mathrm{Numer}\;\mathrm{of}\;\mathrm{CTCs}\;\mathrm{collected}\;\mathrm{in}\;\mathrm{the}\;\mathrm{specific}\;\mathrm{outlet}}{\mathrm{Total}\;\mathrm{number}\;\mathrm{of}\;\mathrm{CTCs}\;\mathrm{in}\;\mathrm{the}\;\mathrm{inlet}}\times 100$$

(15) $$\mathrm{Purity}=\frac{\mathrm{Numer}\;\mathrm{of}\;\mathrm{CTCs}\;\mathrm{collected}\;\mathrm{in}\;\mathrm{the}\;\mathrm{specific}\;\mathrm{outlet}}{\mathrm{Total}\;\mathrm{number}\;\mathrm{of}\;\mathrm{cells}\;\mathrm{in}\;\mathrm{the}\;\mathrm{specific}\;\mathrm{outlets}}\times 100$$

**Figure 6:**
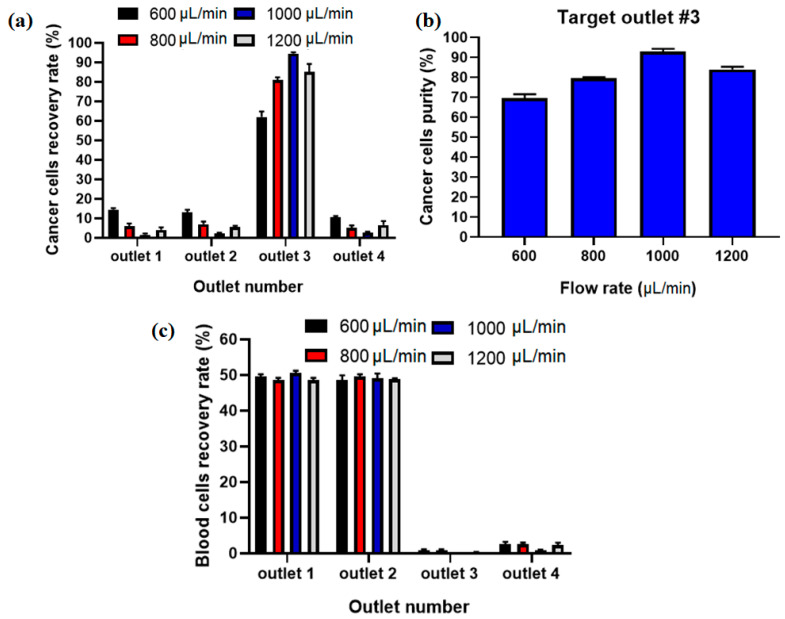
(**a**) Separation efficiency for CTCs at different inlet flow rates. (**b**) Purity of separated CTCs at different flow rates in outlet 3. (**c**) Recovery rate for blood cells at different flow rates and outlets.

The recovery rate/separation efficiency and purity for separated MCF7 cells as well as blood cells for collecting outlets in different flow rates using Equations (8) and (9) were calculated. Figure 6a shows the comparison of the recovery rate of CTCs at different flow rates, and it displays that the proposed hybrid device can separate MCF-7 cells from diluted blood with separation efficiency of ~95% at flow rate of 1000 µL/min. Additionally, Figure 6b shows the purity of captured CTCs, which shows the maximaum purity of ~93% at 1000 µL/min for input sample. At higher flow rates the MCF-7 cells are focused in the middle of the channel in the asymmetric serpentine inertial separator and blood cells (RBCs and WBCs) are focused close to the sidewalls of the inertial separator (as shown in numerical simulation of particle’s movement in inertial section). Figure 6c shows the recovery rate for blood cells in different collecting outlets for different flow rates. Based on this results, the majority of blood cells (~100%) are depleted in the inertial section of the hybrid channel (outlets 1 and 2) before the entrance to the magnetic separator, which leads to higher separation efficiency and purity for CTCs in the magnetic separator as well as in the proposed hybrid device and minimum numbers of blood cells are entered into the target outlet (#3).

**Figure 7:**
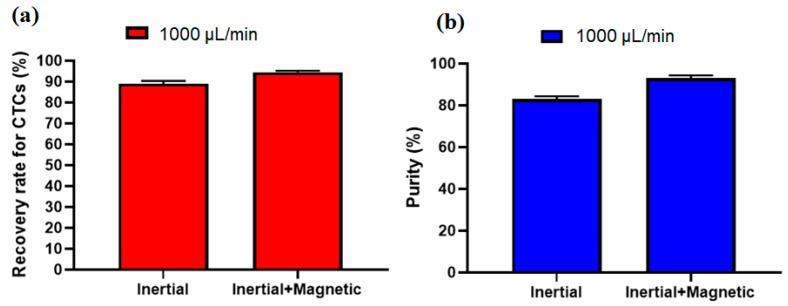
Comparison of the performance of the proposed device in terms of (**a**), recovery rate and (**b**), purity of separated CTCs for the inertial section as well as the inertial-magnetic hybrid device.

To investigate the effect of the inertial section for the proposed hybrid design, another experiment without labeling CTCs with nanoparticles and antibodies was performed at a flow rate of 1000 µL/min (optimal flow rate) and after analyzing the images for collected cells, the recovery rate of 89% and purity of 82% were achieved for inertial section. Figure 7a,b shows the comparison of the inertial and hybrid (inertial + magnetic) cell separation performance in terms of recovery rate and purity at flow rate of 1000 µL/mi. As shown in this figure, the main advantage of using magnetic labeling of CTCs is improving the purity of CTCs, which increased from 82% in the inertial section to 93% in the hybrid (inertial + magnetic) approach. Moreover, the recovery rate also improved about 6% in the hybrid approach compared to the single inertial approach.

**Figure 8:**
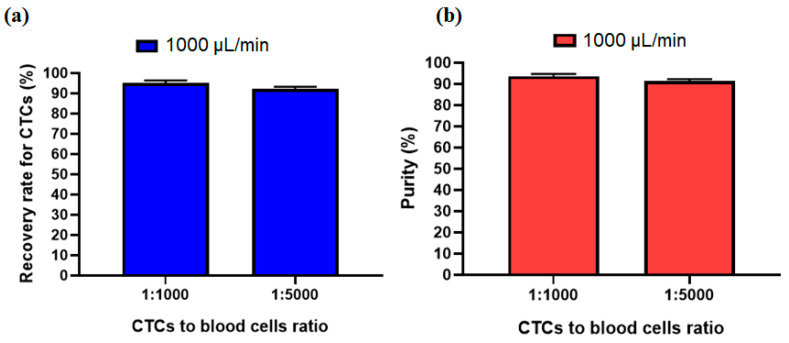
Comparsion of the (**a**) recovery rate and (**b**) purity of separated CTCs at two different ratios of CTC to blood cell numbers.

In order to investigate other ratio for the number of CTCs to blood cells, another experiment was performed to see the effect of the ratio of 1:5000 for CTCs to blood cells number. Figure 8a,b shows that due to the size-based working mechanism of the inertial section the recovery rate and purity of separated cells at an optimal flow rate of 1000 µL/min has no significant change compared to the 1:1000 ratio for CTCs to blood cells number. This shows acceptable performance of our proposed hybrid device for different ratios of CTC to blood cell numbers.

**Figure 9:**
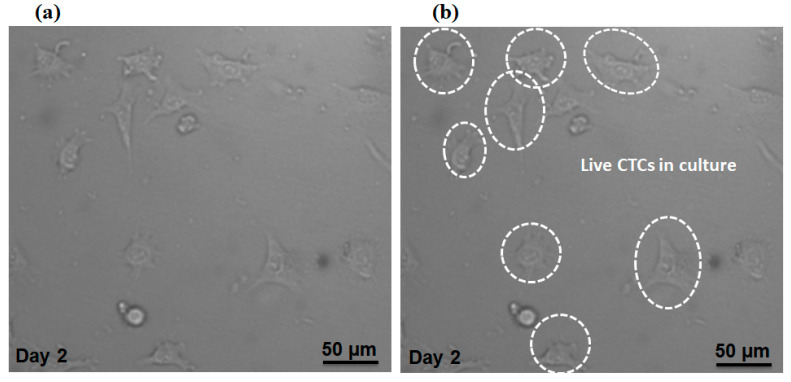
(**a**) Microscopic image for separated CTCs which were cultivated for 48 h. (**b**) Selected cells are live CTCs.

Additionally, we evaluated our proposed device in terms of preserving cell viability for isolated MCF-7 cells. The cell suspension containing MCF-7 cells (without fixing or staining) was injected into the device with the optimal flow rate of the device (1000µL/min). After collecting the MCF-7 cells in the magnetic cell separator unit, the cells were cultivated in a petri dish within the incubator and Figure 9a,b shows the cells at day 2, which indicates the viability of cultivated cells based on their morphology and attachment to the culture substrate. Therefore, the shear stress on the cells due to the fluid flow does not affect the viability of the cells. Therefore, our proposed device able to keep the cell viability for separated MCF-7 cells.

## 4. Limitations and Future Perspectives:

Using our proposed hybrid device, the separation of CTCs from diluted whole blood achieved a high recovery rate and purity. Compared to other the proposed hybrid devices in the literature, our device benefits from high throughput cell separation by the injection of high flow rate and the inertial section in our hybrid device can eliminate ~100% of the blood cells, which helps to achieve a high purity of captured CTCs. Compared to single magnetic cell separation, our proposed hybrid device needs dilution of the blood sample to avoid cell–cell interactions to have an efficient performance for the inertial section of our device, however in the single magnetic device due to large number of blood cells and their interactions, the purity can be affected adversely. Future works with using different dilution ratio can be performed. On the other hand, the linear structure of our proposed device compared to the non-linear devices in literature can take advantage of the parallelization capability of channels to achieve higher throughput applications. Additionally, future works with optimization of the magnetic field in terms of the magnet field can be valuable to achieve optimal magnetic cell separation. Moreover, due to the linear structure of our proposed hybrid device, future work using an array of parallel hybrid devices can be performed to achieve a high throughput cell separation platform with high purity.

Although the magnetically labeled CTCs were collected in the outlet next to the magnet, which shows the effective bonding of the nanoparticle to CTCs, future works using fluorescently tag nanoparticles could provide a better demonstration of binding. Additionally, more characterization about the effect of flow rate on binding efficacy can be performed in future work.

## 5. Conclusions

In this study, the feasibility of a hybrid microfluidic device composed of an inertial separator and a magnetophoretic-based CTCs isolation device was investigated. In the inertial section by designing an asymmetric serpentine device, the majority of RBCs and WBCs were isolated, then the CTCs and the rest of the cells were introduced into the focusing channel and a magnetic separator. The focusing channel was designed to help focus the cells in a stream and the magnetic separator by designing an expansion channel, the CTCs that were conjugated to nanoparticles were separated in presence of a magnetic field. The flow rate of 1000 µL/min led to a maximum recovery rate and purity of 95% and 93%, respectively. Additionally, both separation units, as well as focusing sections, are integrated in a single microchip, which prevents cell loss in the process of cell transfer and a tubing connection between the different cell separation modules. Our proposed integrated three-stage device can be widely employed for precise, fast, and tumor antigen-dependent separation of different target cells with a high efficiency.

## Data Availability

The data presented in this study are available on request from the corresponding author.

## Supplementary Materials

The following are available online at https://www.mdpi.com/article/10.3390/mi12080877/s1, Figure S1: Viscosity of 20 times diluted blood versus shear rate. Figure S2: Fabricated device. (a) photomask, (b) Mold, (c) fabricated hybrid device. Figure S3: (a) Experimental setup, (b) Collecting glass vials. Figure S4: DAPI stained CTCs images at three other flow rates for different outlets. Scale bars are (100 µm).
