# Supplementary material for: Design of a Hybrid Inertial and Magnetophoretic Microfluidic Device for CTCs Separation from Blood
Source: Micromachines (Basel). 2021 Jul 26;12(8):877. doi: 10.3390/mi12080877 (PMC8401779; doi:10.3390/mi12080877)
Supplement: Supplementary file 1 [file micromachines-12-00877-s001.zip › micromachines-1191279-supplementary.pdf]

Supplementary file

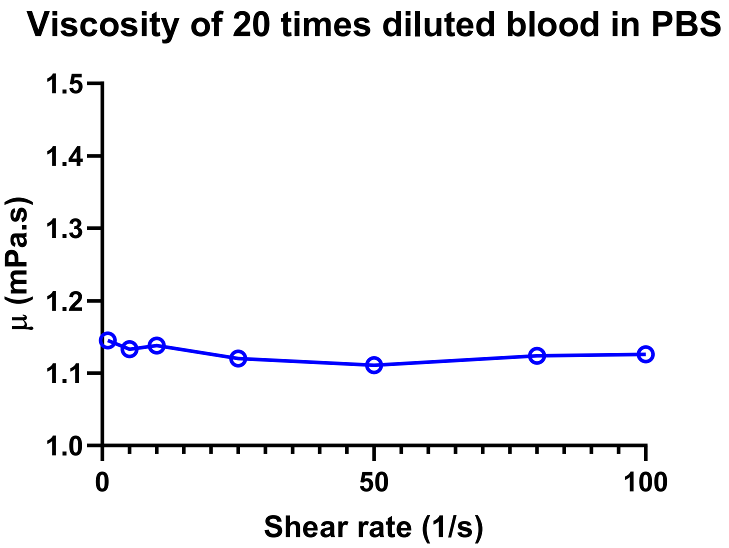

Figure S1. Viscosity of 20 times diluted blood versus shear rate

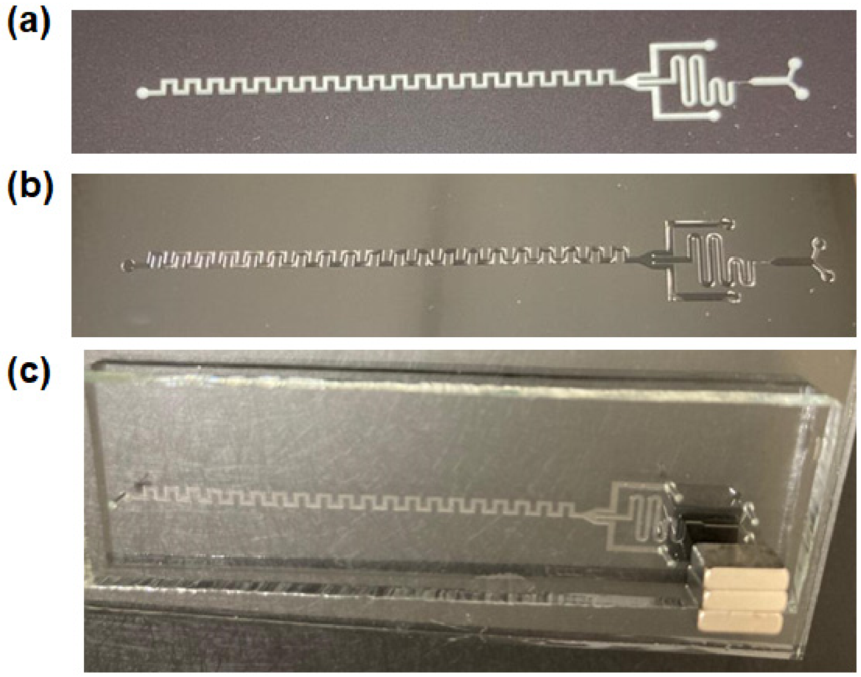

Figure S2. Fabricated device. a) photomask, b) Mold, c) fabricated hybrid device

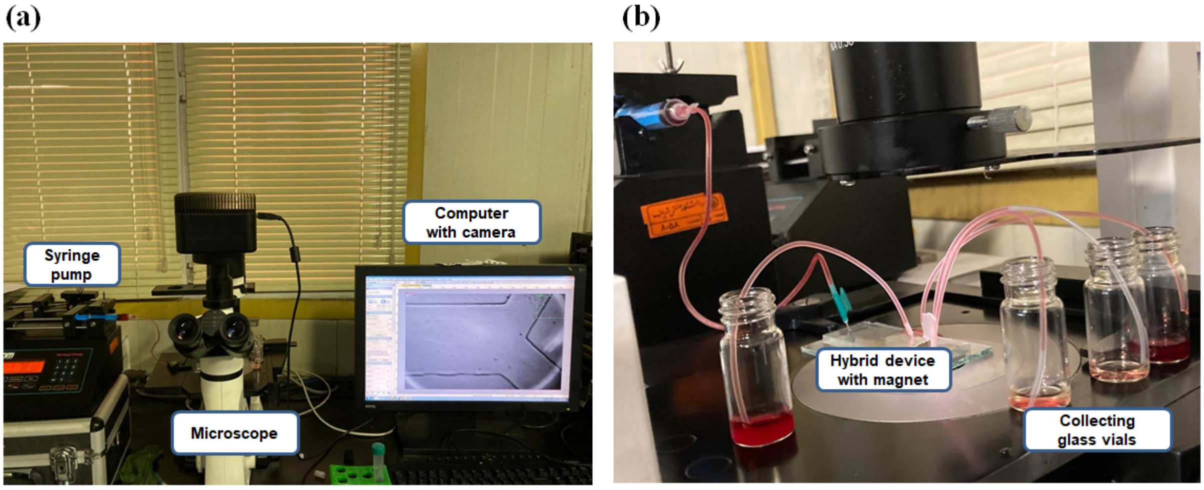

Figure S3. a) Experimental setup, b) Collecting glass vials

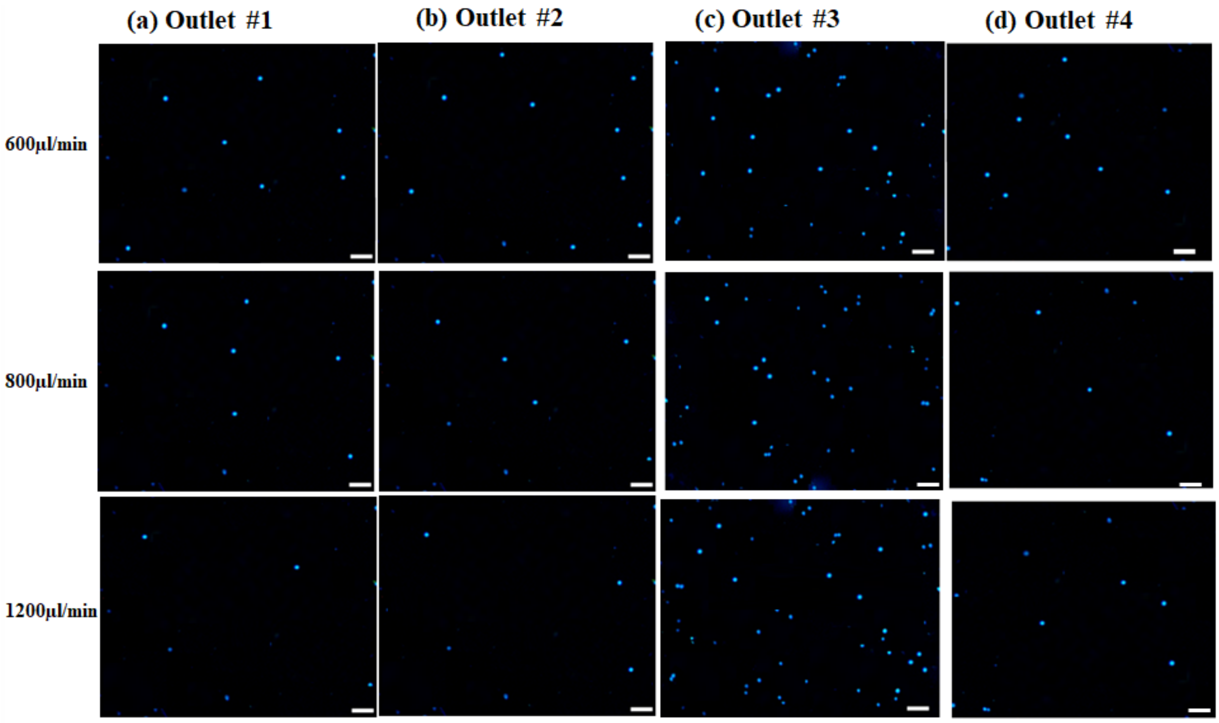

Figure S4. DAPI stained CTCs images at three other flow rates for different outlets. Scale bars are (100 µm).
